# Visualization of Alzheimer’s Disease Related α-/β-/γ-Secretase Ternary Complex by Bimolecular Fluorescence Complementation Based Fluorescence Resonance Energy Transfer

**DOI:** 10.3389/fnmol.2018.00431

**Published:** 2018-11-27

**Authors:** Xin Wang, Gang Pei

**Affiliations:** ^1^State Key Laboratory of Cell Biology, CAS Center for Excellence in Molecular Cell Science, Shanghai Institute of Biochemistry and Cell Biology, Chinese Academy of Sciences, University of Chinese Academy of Sciences, Shanghai, China; ^2^School of Life Science and Technology, and The Collaborative Innovation Center for Brain Science, Tongji University, Shanghai, China

**Keywords:** Alzheimer’s disease, secretase, amyloid precursor protein, bimolecular fluorescence complementation, fluorescence resonance energy transfer

## Abstract

The competitive ectodomain shedding of amyloid-β precursor protein (APP) by α-secretase and β-secretase, and the subsequent regulated intramembrane proteolysis by γ-secretase are the key processes in amyloid-β peptides (Aβ) generation. Previous studies indicate that secretases form binary complex and the interactions between secretases take part in substrates processing. However, whether α-, β- and γ-secretase could form ternary complex remains to be explored. Here, we adopted bimolecular fluorescence complementation in combination with fluorescence resonance energy transfer (BiFC-FRET) to visualize the formation of triple secretase complex. We show that the interaction between α-secretase ADAM10 and β-secretase BACE1 could be monitored by BiFC assay and the binding of APP to α-/β-secretase binary complex was revealed by BiFC-FRET. Further, we observed that γ-secretase interacts with α-/β-secretase binary complex, providing evidence that α-, β- and γ-secretase might form a ternary complex. Thus our study extends the interplay among Alzheimer’s disease (AD) related α-/β-/γ-secretase.

## Introduction

Amyloid plaques primarily composed of amyloid-β peptides (Aβ) have been identified to be one of the major hallmarks of Alzheimer’s disease (AD) (Masters et al., 1985; Huang and Mucke, 2012). Amyloid cascade hypothesis suggest that the imbalance between Aβ production and clearance might be the central event and often initiating factor in AD pathogenesis (Hardy and Selkoe, 2002; Selkoe and Hardy, 2016). Aβ peptides are derived from amyloid-β precursor protein (APP) through sequential proteolysis by β- and γ-secretase (Vassar, 1999; De Strooper, 2003; Blennow et al., 2006). In the non-amyloidogenic pathway, α-secretase competes with β-secretase in APP ectodomain shedding and cleaves APP within Aβ domain, thus precludes Aβ production (Skovronsky et al., 2000; Colombo et al., 2013). For AD treatment, researchers have put much effort to reduce Aβ generation through inhibiting or modulating β- or γ-secretase activities (De Strooper et al., 2010; Yan and Vassar, 2014; MacLeod et al., 2015). However, most of the chemicals targeting the secretases showed side effects in clinical trials due to the diverse substrates of secretases (Graham et al., 2017; Voytyuk et al., 2018). Therefore, it is crucial to gain insights into the molecular mechanism of secretases activities in APP processing.

Previous studies have shown that β- or γ-secretase could form dimers to mediate APP processing (Schroeter et al., 2003; Cervantes et al., 2004; Schmechel et al., 2004; Westmeyer et al., 2004; Jin et al., 2010; Liebsch et al., 2017). Besides, α- and β-secretase were reported to interact with γ-secretase physically and functionally (Hattori et al., 2002; Hebert et al., 2003; Chen et al., 2015; Cui et al., 2015), and a new model that proteases may form functional complexes and execute sequential cleavage efficiently was proposed (Chen et al., 2015). Recently, we reported the interaction between α- and β-secretase in neurons, supporting the concept of multiprotease complex (Wang et al., 2018). To further explore the relationship among AD-related secretases, we sought to investigate whether α-, β-and γ-secretase could form ternary complex. Fluorescence resonance energy transfer (FRET) and bimolecular fluorescence complementation (BiFC) have been applied to visualize β- or γ-secretase dimerization, interactions between secretases or between γ-secretase subunits (Meckler and Checler, 2014; Cui et al., 2015; Liebsch et al., 2017; Wang et al., 2018). We exploit BiFC based FRET (BiFC-FRET) assay to visualize ternary secretase complex in intact cells (Shyu et al., 2008; Midde et al., 2015). Here, we show that the specific interaction between α-secretase ADAM10 and β-secretase BACE1 could be observed using BiFC assay. Taking advantage of BiFC-FRET, we found that APP binds to α-/β-secretase binary complex. Further, we provide evidence that α-, β- and γ-secretase might form a ternary complex utilizing BiFC-FRET. Besides, α-/β-/γ-secretase BiFC-FRET was influenced by APP, suggesting that α-/β-/γ-secretase ternary complex might be functional.

## Materials and Methods

### Antibodies, Plasmids and Chemicals

Immunoblotting and immunostaining were performed with the following antibodies: anti-ADAM10 (Ab1997, Abcam); anti-BACE1 (MAB5308, EMD Millipore Corporation); anti-Flag (F3156, Sigma); anti-actin (A2066, Sigma); anti-GM130 (610822, BD Transduction Laboratories); anti-EEA1 (610457, BD Transduction Laboratories).

cDNA sequences of mCherry, human BACE1, BACE1 N-terminal peptide LT52 (amino acids 43–94) and the scramble control peptide LT52-S were cloned into pcDNA3 or pcDNA4 vector. The cDNA sequences of human Aph1aL, Nct, PS1 and Pen2 were subjected to codon optimization (Life Technologies) and cloned into pMlink vector. pMlink-Aph1aL, pMlink-Nct and pMlink-Pen2 were combined by the LIC method to generate pMLink-nicastrin-Pen2-Aph1aL as reported previously (Lu et al., 2014). For BiFC and FRET constructs, the C- or N-terminal fragments of Venus (with I152L mutation), or mTurquoise2 was fused to the C-terminus of ADAM10 or BACE1 or APP with a linker (GSGGGGSGGGGS) and cloned into pMlink vector, and mTurquoise2 was fused to the N-terminus of PS1 and cloned into pMlink vector. We performed site-directed mutagenesis using an overlapping PCR strategy to generate APPαM, APPβM and APPαβM. Flag tag were fused to the C-terminus of wild-type and mutated APP.

Secretase inhibitor GI254023X (3995, TOCRIS), BACE1 inhibitor-IV (BSI-IV; 565788, Calbiochem) and L685, 458 (L1790, Sigma) were dissolved in DMSO before use.

### Cell Culture and Transfection

HEK 293 cells were obtained from ATCC and cultured in Minimum Essential Medium (MEM) supplemented with 10% FBS at 37°C under 5% CO_2_. Neuro-2a cells were obtained from ATCC and cultured in MEM supplemented with 10% FBS at 37°C under 5% CO_2_, and transfected in MEM supplemented with 0.5% FBS to allow differentiation. HEK 293/APPswe cells were stably transfected with APP Swedish mutant (APPswe-HA).

Cells were plated onto 12-well plates (for Western blots) or glass coverslip contained 12-well plates (for co-localization, BiFC and FRET analysis), and transfected using Effectene Transfection Reagent (QIAGEN, 301427). For BiFC detection, cells were transfected with 0.1–0.2 μg of BiFC probes per well of 12-well plate. For BiFC-FRET detection, cells were transfected with 0.1 μg BiFC probes, 0.05 μg of Tur-PS1, 0.1 μg of pMlink-Nct-Aph1aL-Pen2 and with or without 0.075 μg of APP-flag constructs per well of 12-well plate.

### Bimolecular Fluorescence Complementation (BiFC)

HEK 293 cells were transfected with BiFC constructs for 16 h, fixed with PBS/2% paraformaldehyde, stained with Hoechst 33342 and then visualized by confocal microscopy (LAS SP8; Leica) using 20×/0.75 NA IMM or 63×/1.40 NA oil objective (Leica). Imaging conditions were used: Hoechst 33342 (excitation: 405 nm, emission: 425–465 nm), and Venus (excitation: 514 nm, emission: 525–600 nm). Images acquired under 20× objective were used for quantification.

To monitor the expression of BiFC constructs in cells, HEK 293 cells used for Western blots analysis were plated and transfected at the same time with the same transfection mix as BiFC analysis. HEK 293 cells were also harvested 16 h after transfection for immunoblots analysis.

The fluorescence intensity of Venus and total cell number in 20× images and protein expression analyzed by Western blots were quantified respectively using ImageJ software, and relative BiFC signal was calculated as: Venus fluorescence intensity/(total cell number × expression of ADAM10 and BACE1).

### Western Blots Analysis

After transfection, HEK 293 cells were lysed in 1× loading buffer. Protein samples were resolved on 10% or 12% SDS-PAGE and transferred to nitrocellulose membranes. After blocked by dried skimmed milk in TBST, membranes were incubated with primary antibodies, washed three times and incubated with HRP-conjugated secondary antibodies. The membranes were incubated with ECL substrates (Bio-rad) and then scanned by Tanon-5200 system. The quantitative analysis was carried out by measuring the gray value of the blot bands using ImageJ software.

### Immunofluorescence Staining Analysis

For lysosome staining, cells were incubated with LysoTracker (Molecular probes) for 30 min and fixed with PBS/2% paraformaldehyde followed by Hoechst 33342 staining. For Cis-Golgi complex and early endosome staining, cells were fixed with PBS/2% paraformaldehyde, permeabilized, and blocked with PBS/0.1% Saponin/2% BSA followed by incubation with primary antibodies at room temperature for 2 h. After washed with PBS/0.1% Saponin/2% BSA, cells were incubated with Alex647-labeled secondary antibodies for 1 h and followed by Hoechst 33342 staining in the dark at room temperature. Then cells were mounted on slides for image acquisition under a LAS SP8 confocal microscope (Leica) with a 63×/1.40 NA oil objective (Leica).

### Acceptor Photobleaching Fluorescence Resonance Energy Transfer (FRET) Measurement

For BiFC-FRET assay, 16 h (HEK 293 cells) or 24 h (neuro-2a cells) after transfection, cells were fixed with PBS/2% paraformaldehyde, washed and mounted on slides for FRET detection. Samples were then subjected to acceptor photobleaching FRET imaging under a confocal microscope (LAS SP8; Leica) with a 63×/1.40 NA oil objective (Leica) as described previously (Wang et al., 2015). Image acquisition, registration, background subtraction, and data analyses were performed with Leica Application Suite Advanced Fluorescence (LAS AF) software. Imaging conditions were set up manually: mTurquoise2 (excitation: 458 nm, emission: 465–505 nm), and Venus (excitation: 514 nm, emission: 525–600 nm). Photobleach was performed using acceptor excitation light and the similar bleach efficiency (~80%–90%) was achieved. Images of donor and acceptor channels were acquired pre- and post-bleach, respectively. FRET efficiency was calculated as percentage of enhancement in donor fluorescence (f) after acceptor photobleaching: *E* = 1 − f[CFP(pre)]/f[CFP(post)]. Five non-bleached regions were selected and the average value was used to correct the FRET efficiency of photobleached region.

### Statistical Analysis

All experiments were repeated at least three times and data are presented as Mean ± SEM and analyzed by GraphPad Prism 6.01 (San Diego, CA, USA). Unpaired Student’s *t*-test was used for the comparisons between two groups. One-way analysis of variance (ANOVA) followed Bonfferoni’s multiple comparisons test was used for the comparisons among more than two groups.

## Results

### BiFC Reveals α-/β-Secretase Complex

**Figure 1:**
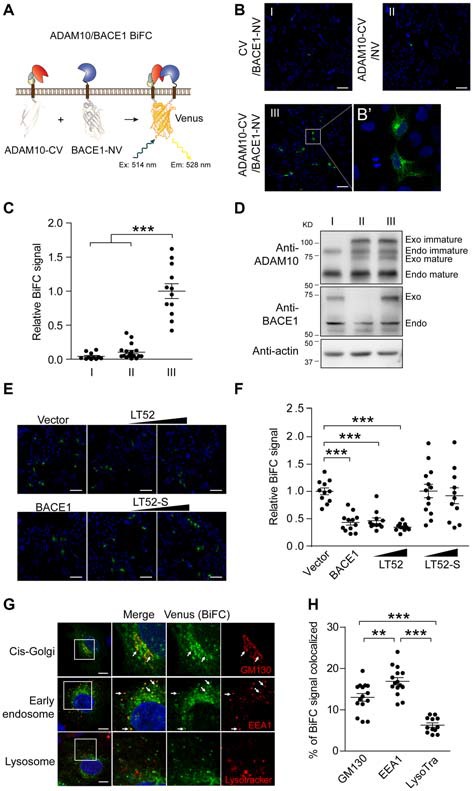
Bimolecular fluorescence complementation (BiFC) reveals α-/β-secretase complex.** (A)** Schematic for ADAM10-CV and BACE1-NV constructs. The C-fragment and N-fragment of Venus was fused to the C-terminal of ADAM10 or BACE1 respectively. CV, C-fragment of Venus; NV, N-fragment of Venus. **(B)** BiFC signal between ADAM10 and BACE1. HEK 293 cells were transfected with CV and BACE1-NV **(I)** or ADAM10-CV and NV **(II)**, or ADAM10-CV and BACE1-NV **(III)**, and fixed 16 h later for BiFC detection. The BiFC signals were examined by confocal imaging under 20× objective, scale bar: 100 μm or **(B’)** under 63× objective. **(C)** Quantification of fluorescence intensity of ADAM10/BACE1 BiFC signals in **(B)**. Images under 20× objective were evaluated, *N* = 11–18 per condition. A one-way analysis of variance (ANOVA) with Bonferroni’s multiple comparisons was used. ***p < 0.001. **(D)** Western blots show the expression of ADAM10-CV and BACE1-NV in **(B)**. Specific antibodies for ADAM10 or BACE1 were used to detect the endogenous and Venus fragment tagged ADAM10 or BACE1. Immunoblots show the near endogenous expression level of ADAM10-CV and BACE1-NV. **(E)** ADAM10/BACE1 BiFC signal is attenuated by LT52. HEK 293 cells were transfected with ADAM10-CV, BACE1-NV and designated plasmids, and fixed 16 h later. The expression of BACE1 or LT52 attenuates ADAM10/BACE1 BiFC signal, while LT52-S shows little effect. Scale bar: 100 μm. **(F)** Quantification of ADAM10/BACE1 BiFC signals in **(E)**. *N* = 11–13 per condition. A one-way ANOVA with Bonferroni’s multiple comparisons was used. ***p < 0.001. **(G)** Subcellular location of ADAM10/BACE1 BiFC signal. HEK 293 cells were transfected with ADAM10-CV and BACE1-NV for 16 h, and incubated with LysoTracker, or fixed and subjected to immunofluorescence staining for GM130 or EEA1. Scale bar: 10 μm. The arrows point to the co-localized regions. **(H)** Quantification of localization of ADAM10/BACE1 BiFC in cis-Golgi, early endosome and lysosome. *N* = 12–15 per condition. A one-way ANOVA with Bonferroni’s multiple comparisons was used. **p < 0.01, ***p < 0.001.

The pairwise interactions of α-, β- and γ-secretase have been previously reported, we developed BiFC based FRET (BiFC-FRET) assay to visualize ternary complex in intact cells. First, we studied the interaction between α-secretase ADAM10 and β-secretase BACE1 using BiFC assay. We engineered BiFC constructs by fusing complementary C-fragment (CV) or N-fragment (NV) of Venus to the C-terminal of ADAM10 or BACE1 to generate fusion protein ADAM10-CV and BACE1-NV (Figure 1A). In HEK 293 cells stably overexpressing APP, we found that Venus fragment tagged ADAM10 and BACE1 possess catalytic activities to cleave APP (Supplementary Figure 1A). We assessed BiFC signal 16 h after transfection to diminish the effect of BiFC complex accumulation. Venus BiFC fluorescence was scarcely seen in cells transfected with BACE1-NV and only CV fragment or in cells transfected with ADAM10-CV and only NV fragment (Figures 1BI,II), while was more frequently observed in cells transfected with ADAM10-CV and BACE1-NV (Figure 1BIII, quantified in Figure 1C). Besides, we confirmed the expression levels of ADAM10-CV and BACE1-NV to be near those of endogenous proteins to avoid artificial interaction caused by robust expression (Figure 1D). Reversely, Venus fluorescence in cells transfected with ADAM10-NV and BACE1-CV (Supplementary Figure 1BIII, immunoblots analysis shown in Supplementary Figure 1C) was also significantly stronger than those in cells transfected with ADAM10-NV and only CV fragment or BACE1-CV and only NV fragment (Supplementary Figures 1BI,II, quantified in Supplementary Figure 1D). Although Venus fluorescence in cells transfected with ADAM10-NV and BACE1-CV was relative weaker than that in cells transfected with ADAM10-CV and BACE1-NV, these data suggests that ADAM10/BACE1 interaction was more favorable to the complementation of ADAM10-CV and BACE1-NV. To further confirm the BiFC efficiency, we examined ADAM10/BACE1 BiFC signal with the expression of mCherry as a control. As shown in Supplementary Figures 1E,F, ADAM10/BACE1 BiFC signal could be observed in the majority of mCherry-expressing cells, indicating that the interaction between ADAM10 and BACE1 bring the two nonfluorescent halves of Venus together and generate BiFC signal efficiently.

To further testify the BiFC fluorescence is caused by ADAM10/BACE1 interaction, we examined the effect of LT52 (Supplementary Figure 1G), a peptide that was reported to attenuate ADAM10/BACE1 interaction (Wang et al., 2018), on ADAM10/BACE1 BiFC signal. As shown in Figure 1E, the expression of LT52 led to significant reduction of BiFC fluorescence (quantified in Figure 1F), and showed much less effect on ADAM10-CV and BACE1-NV expression level (Supplementary Figure 1H). However, the expression of scrambled peptide LT52-S (Supplementary Figure 1G) that has no influence on ADAM10/BACE1 interaction, showed little effect on BiFC fluorescence (Figures 1E,F). These data suggest that the BiFC recapitulated ADAM10/BACE1 specific interaction.

Next, we inquired into the ADAM10/BACE1 BiFC by high-resolution imaging. As shown in Figure 1B′, Venus BiFC fluorescence in cells transfected with ADAM10-NV and BACE1-CV largely distributed in punctate pattern. Then we examined the subcellular localization of ADAM10/BACE1 BiFC signal. HEK 293 cells transfected with ADAM10-CV and BACE1-NV were stained with specific antibody for Golgi complex marker GM130 or early endosome marker EEA1, or incubated with lysosome probe LysoTracker. As shown in Figure 1G, ADAM10/BACE1 BiFC signal co-localized with GM130, while the BiFC puncta showed more significant co-localization with EEA1 but little with LysoTracker (quantified in Figure 1H).

Together, these data indicate that the interaction between ADAM10 and BACE1 could be monitored by BiFC assay in intact cells.

### APP α-/β-Cleavage Sites Mutant Enhances BiFC Signal of α-/β-Secretase

**Figure 2:**
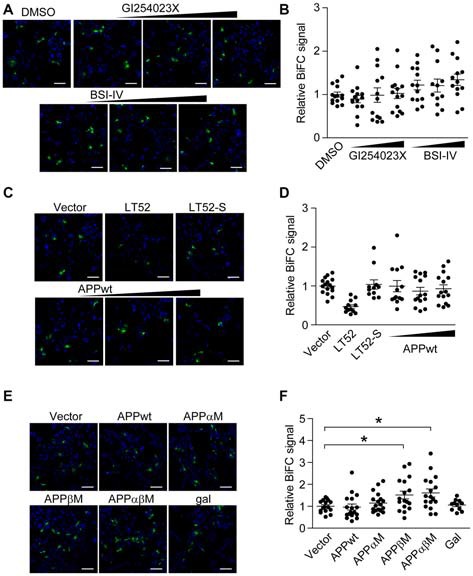
Amyloid-β precursor protein (APP) α-/β- cleavage sites mutant enhances BiFC signal of α-/β-secretase.** (A,B)** Secretases inhibitors do not affect ADAM10/BACE1 BiFC signal. HEK 293 cells were transfected with ADAM10-CV and BACE1-NV for 8 h, and treated with 0.3 μM or 1 μM or 3 μM α-secretase inhibitor GI254023X, or treated with 0.1 μM or 0.3 μM or 1 μM β-secretase inhibitor BSI-IV. Eight hours after treatment, cells were fixed for BiFC detection **(A)** scale bar: 100 μm. **(B)** Treatment with GI254023X or BSI-IV showed little effect on ADAM10/BACE1 BiFC signal. Images under 20× objective were evaluated, *N* = 12–14 per condition. A one-way ANOVA with Bonferroni’s multiple comparisons was used. **(C,D)** Wild-type APP (APPwt) do not affect ADAM10/BACE1 BiFC signal. HEK 293 cells were transfected with ADAM10-CV, BACE1-NV and designated plasmids, and fixed 16 h later for BiFC detection **(C)** scale bar: 100 μm. **(D)** Expression of APPwt showed little effect on ADAM10/BACE1 BiFC signal. *N* = 11–16 per condition. A one-way ANOVA with Bonferroni’s multiple comparisons was used. **(E,F)** APP cleavage site mutants affect ADAM10/BACE1 BiFC signal. HEK 293 cells were transfected with ADAM10-CV, BACE1-NV and APP for 16 h and fixed for BiFC detection **(E)** scale bar: 100 μm. **(F)** Expression of APPwt or APPαM showed little effect on ADAM10/BACE1 BiFC signal, while expression of APPβM or APPαβM enhanced ADAM10/BACE1 BiFC signal. *N* = 16–19 per condition. A one-way ANOVA with Bonferroni’s multiple comparisons was used. *p < 0.05.

We asked whether ADAM10 and BACE1 inhibitors affect ADAM10/BACE1 BiFC signal. Cells transfected with ADAM10-CV and BACE1-NV were treated with ADAM10 inhibitor GI254023X or BACE1 inhibitor BSI-IV, and we found that these inhibitors showed little effect on ADAM10/BACE1 BiFC signal (Figures 2A,B and Supplementary Figure 2A).

Next, we examined whether the sharing substrate APP has an impact on ADAM10/BACE1 BiFC signal. HEK 293 cells were transfected with ADAM10-CV, BACE1-NV and wild-type APP (APPwt; Supplementary Figure 2B). As shown in Figures 2C,D, the expression of APPwt did not affect ADAM10/BACE1 BiFC signal. In addition, the expression of α-secretase cleavage site mutated APP (APPαM; Yamada and Kobayashi, 1995) also showed little effect on ADAM10/BACE1 BiFC signal (Figures 2E,F). However, we observed that the expression of β-secretase cleavage site mutated APP (APPβM; Zhou et al., 2011) and α-/β-secretase cleavage sites mutated APP (APPαβM) enhanced ADAM10/BACE1 BiFC signal significantly (Figure 2E, quantified in Figure 2F, expression analyzed in Supplementary Figure 2C), indicating that APPβM and APPαβM might stabilize the association between α- and β-secretase. As substrate binding usually induces the conformational changes of enzyme, the data suggest that APP might bind to α-/β-secretase complex.

### Visualization of APP/α-/β-Secretase Ternary Complex Using BiFC-FRET

**Figure 3:**
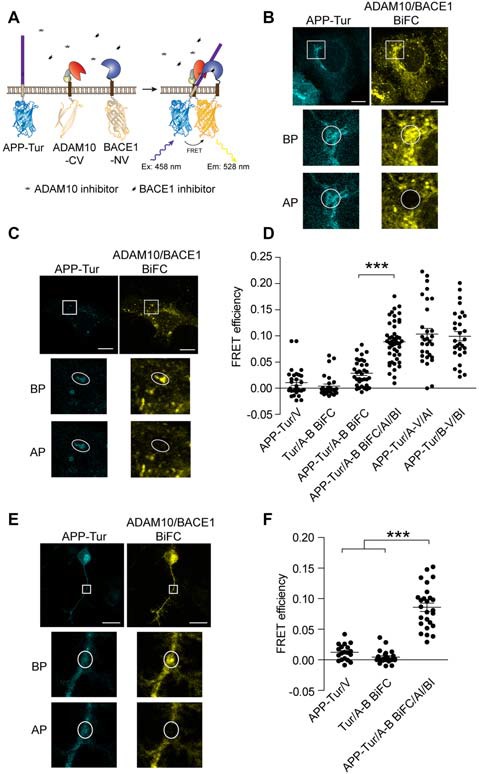
Visualization of APP/α-/β-secretase ternary complex using BiFC-fluorescence resonance energy transfer (FRET).** (A)** Schematic for ADAM10-CV, BACE1-NV and APP-mTurquoise2 (APP-Tur) constructs. mTurquoise2 were fused to the C-terminal of APPwt. **(B,C)** Representative images of APP/ADAM10/BACE1 BiFC-FRET analysis in **(D)**. Scale bar: 10 μm. HEK 293 cells were transfected with ADAM10-CV, BACE1-NV and APP-Tur for 12 h, and treated with DMSO **(B)** or 3 μM GI254023X and 1 μM BSI-IV **(C)** for 4 h before fixation. **(D)** FRET analysis between APP-Tur and ADAM10/BACE1 BiFC particles. HEK 293 cells were transfected with designated plasmids for 16 h, and fixed for FRET analysis. AI: treated with 3 μM α-secretase inhibitor GI254023X, BI: treated with 1 μM β-secretase inhibitor BSI-IV for 4 h. BP: before photobleach, AP: after photobleach. *N* = 28–46 per condition. A one-way ANOVA with Bonferroni’s multiple comparisons was used. ***p < 0.001. **(E)** FRET analysis between APP-Tur and ADAM10/BACE1 BiFC particles in differentiated neuro-2a cells. Neuro-2a cells were transfected for 16 h followed by treatment with GI254023X and BSI-IV for 8 h, and fixed for FRET analysis. Scale bar: 20 μm. **(F)** Quantification of FRET analysis in **(E)**. *N* = 19–26 per condition. A one-way ANOVA with Bonferroni’s multiple comparisons was used. ***p < 0.001.

We sought to investigate whether APP incorporates into binary α-/β-secretase complex further. In order to visualize the formation of APP/α-/β-secretase ternary complex directly, we utilized BiFC-FRET assay. If APP binds to α-/β-secretase complex, cyan fluorescent protein mTurquoise2 (Tur) tagged APP would be close to reconstituted Venus tagged α-/β-secretase complex, then fluorescent energy transfer occurs between mTurquoise2 and Venus (Figure 3A). We transfected HEK 293 cells with APP-Tur, ADAM10-CV and BACE1-NV, and observed that the co-localization of APP-Tur and ADAM10/BACE1 BiFC signal was mainly seen in the stacks structure adjacent to nucleus (Figure 3B). We adopted acceptor photobleaching method to detect the FRET efficiency between APP-Tur and ADAM10/BACE1 BiFC signal, and a low FRET efficiency of 2.86% was obtained (Figures 3D, APP-Tur/A-B BiFC). However, when treated with GI254023X and BSI-IV, the co-localization of APP-Tur and ADAM10/BACE1 BiFC signal was observed in vesicles scattered throughout the cytoplasm (Figure 3C), and the FRET efficiency between APP-Tur and ADAM10/BACE1 BiFC signal was significantly higher to be 8.85% (Figure 3D, APP-Tur/A-B BiFC/AI/BI). Besides, we assessed the FRET efficiencies between APP and ADAM10 or BACE1 simultaneously (Supplementary Figures 3A,B), and the FRET efficiencies of 10.34% and 9.93% were detected, respectively (Figure 3D, APP-Tur/A-V/AI and APP-Tur/B-V/BI). These results indicate that similar with its binding to α- or β-secretase, APP interacts with α-/β-secretase complex and might form a ternary complex with α-/β-secretase.

To explore whether APP/α-/β-secretase complex form in physiological conditions, we transfected differentiated neuro-2a cells with APP-Tur, ADAM10-CV and BACE1-NV. As shown in Figures 3E,F, when treated with GI254023X and BSI-IV, we observed that FRET could also occur between APP and ADAM10/BACE1 BiFC signal, suggesting that APP, α- and β-secretase could form ternary complex in neuronal cells.

### Visualization of Ternary Complex of α-/β-Secretase With γ-Secretase Using BiFC-FRET

**Figure 4:**
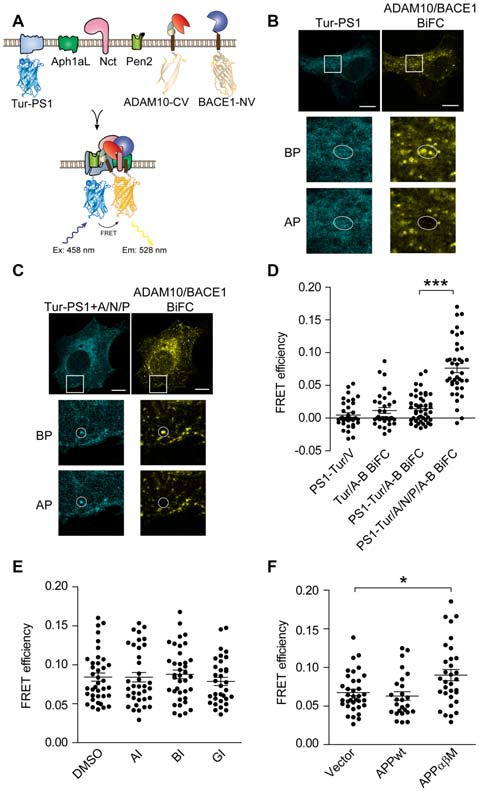
Visualization of ternary complex of α-/β-secretase with γ-secretase using BiFC-FRET. **(A)** Schematic for ADAM10-CV, BACE1-NV and mTurquoise2-PS1 (Tur-PS1) constructs. mTurquoise2 were fused to the N-terminal of PS1. **(B,C)** Representative images of PS1/ADAM10/BACE1 BiFC-FRET in **(D)**. Scale bar: 10 μm. HEK 293 cells were transfected with ADAM10-CV/BACE1-NV/Tur-PS1 **(B)** or ADAM10-CV/BACE1-NV/Tur-PS1/ Aph1aL (A)/Nct (N)/Pen2 (P); **(C)** for 16 h and fixed for FRET analysis. **(D)** FRET analysis of α-/β-/γ-secretase ternary complex. HEK 293 cells were transfected with designated plasmids for 16 h, and fixed for FRET analysis. *N* = 34–46 per condition. A one-way ANOVA with Bonferroni’s multiple comparisons was used. ***p < 0.001. **(E)** Secretases inhibitors do not affect α-/β-/γ-secretase BiFC-FRET. HEK 293 cells were transfected with ADAM10-CV/BACE1-NV/Tur-PS1/A/N/P for 8 h and treated with 3 μM α-secretase inhibitor GI254023X (AI) or 1 μM β-secretase inhibitor BSI-IV (BI) or 1 μM γ-secretase inhibitor L685, 458 (GI) for another 8 h. *N* = 33–36 per condition. A one-way ANOVA with Bonferroni’s multiple comparisons was used. **(F)** APP α-/β- cleavage sites mutant enhanced α-/β-/γ-secretase BiFC-FRET. HEK 293 cells were transfected with ADAM10-CV/BACE1-NV/Tur-PS1/A/N/P and designated plasmids for 16 h, and fixed for FRET analysis. *N* = 27–36 per condition. A one-way ANOVA with Bonferroni’s multiple comparisons was used. *p < 0.05.

Taking advantage of BiFC-FRET to visualize ternary complex formation, we further explored whether α-, β- and γ-secretases form a triple protease complex. If γ-secretase binds to α-/β-secretase complex, PS1 would be close to α-/β-secretase complex, and fluorescent energy transfer may occur between mTurquoise2 and reconstituted Venus tagged on PS1 and α-/β-secretase complex, respectively (Figure 4A). We transfected HEK 293 cells with Tur-PS1, ADAM10-CV and BACE1-NV, and observed little co-localization of reticular distributed Tur-PS1 and ADAM10/BACE1 BiFC signal (Figure 4B). Besides, the FRET efficiency between Tur-PS1 and ADAM10/BACE1 BiFC signal was low in this case (Figure 4D, PS1-Tur/A-B BiFC). However, in cells transfected with Tur-PS1 and γ-secretase subunits Aph1aL/Nct/Pen2 (A/N/P), Tur-PS1 distributed in punctate pattern and showed obvious co-localization with ADAM10/BACE1 BiFC signal (Figure 4C). Meanwhile, significant FRET efficiency between Tur-PS1 and ADAM10/BACE1 BiFC signal was detected when A/N/P were expressed (Figure 4D, PS1-Tur/A/N/P/A-B BiFC). Our previous report has shown that Tur-PS1 could incorporate into γ-secretase complex and possess catalytic activity when expressed with γ-secretase subunits (Wang et al., 2015). These result suggested that holo-γ-secretase might form ternary complex with α- and β-secretase.

To test whether secretase inhibitors affect α-/β-/γ-secretase ternary complex, HEK 293 cells transfected with Tur-PS1/A/N/P and ADAM10/BACE1 BiFC constructs were treated with α-secretase inhibitor GI254023X (AI), β-secretase inhibitor BSI-IV (BI) or γ-secretase inhibitor L685, 458 (GI), but little effect was observed (Figure 4E). Next, we examined whether APP bind to α-/β-/γ-secretase ternary complex, we examined the effect of APP on α-/β-/γ-secretase BiFC-FRET signal. HEK 293 were transfected with BiFC-FRET probes and APPwt or APPαβM, and we observed that APPwt showed little effect on BiFC-FRET efficiency, while APPαβM enhanced it (Figure 4F), indicating that APP might bind to α-/β-/γ-secretase ternary complex and the ternary complex might be functional.

## Discussion

Previous studies showed that FRET could be applied to investigate interactions between α- and β-secretase, and between β- and γ-secretase. In this study, we corroborate the interaction between α- and β-secretase using BiFC assay. By comparing BiFC signals in cells expressing ADAM10-CV and BACE1-NV or BACE1-CV and ADAM10-NV, and examining the effect of ADAM10/BACE1 interaction-interfering peptide, we confirmed that the BiFC signals are primarily generated from specific interaction between ADAM10 and BACE1. Further, we provide evidence for the first time that APP and γ-secretase could form ternary complex with α-/β-secretase complex taking advantage of the combination of BiFC and FRET, and reinforce the idea that secretases may form functional multiprotease complex (Chen et al., 2015). However, the mechanism about the formation of these substrate-multiprotease complexes needs to be explored further. Our previous study showed that the extracellular domains of ADAM10 and BACE1 directly interact (Wang et al., 2018), thus it is possible that ADAM10 might bind directly to BACE1 and then APP or γ-secretase would attach to the complex. On the other hand, our previous study also suggests that ADAM10 and BACE1 partially co-localize, and the interaction between APP and BACE1 was observed in many studies (Kinoshita et al., 2006; Das et al., 2013, 2016). Therefore, it is also possible that APP might directly interact with BACE1 or ADAM10 then the other enzyme bind to the complex. It could be speculated that different multiprotease complexes exist according to the reported studies.

BiFC assay enables direct visualization of protein interactions without any complicated post analysis, which is also favorable to determine the subcellular locations where interactions happen (Kerppola, 2006). We investigated the location of ADAM10/BACE1 binary complex and observed the BiFC signals were primarily located in early endosomes, which is much consistent with previous reports about the sites of APP processing (Sannerud et al., 2011; Choy et al., 2012). Although it is supposed that ADAM10 cleaves APP at plasma membrane (Xu et al., 2009), ADAM10 was also reported to remove from plasma membrane through clathrin-mediated endocytosis (Marcello et al., 2013), locate in early endosome (Escrevente et al., 2008) and exert proteolysis activity at endosomal pH (Mathews et al., 2010). Further, whether ADAM10/BACE1 binary complex exists in plasma membrane or other activity subcellular organelles needs to be explored.

To probe into the formation of secretases complex further, we examined the effects of α- and β-secretase inhibitors on ADAM10/BACE1 BiFC signal. However, little effect was observed, which is in accordance with our previous results that α-secretase inhibitor TAPI had no influence on ADAM10/BACE1 FRET efficiency (Wang et al., 2018), indicating that the binding of inhibitor to the catalytic pocket might not affect ADAM10/BACE1 interaction. Previous study also reported that the interaction between β- and γ-secretase was not affected by classic β- and γ-secretase inhibitors (Cui et al., 2015). These data suggest that the conformational changes of secretases induced by the binding of inhibitors may be insufficient to alter the interactions between them, or maybe the catalytic pockets were not much crucial to the interactions. Although much effort has been put to reduce Aβ generation to cure AD, most of the inhibitors of β-secretase or γ-secretase failed in the clinical trials due to their side effects (Graham et al., 2017; Voytyuk et al., 2018). Targeting the APP-secretase or secretase-secretase complex might be a novel therapeutic strategy, but the classic secretase inhibitors don’t seem to work in this way. To discover small molecules that interfere with the interactions between secretases or between APP and secretase might modulate APP cleavage and Aβ generation.

Although our previous study showed that APP processing was hardly affected by ADAM10/BACE1 interaction interference, we observed the binding of APP to α-/β-secretase binary complex by BiFC-FRET. The FRET efficiency between APP and α-/β-secretase binary complex is low in perinuclear structure, consistent with the notion that ADAM10 and BACE1 might not be active in this compartment (Capell et al., 2000; Yan et al., 2001; Noy et al., 2016). However, when cells were treated with ADAM10 and BACE1 inhibitors, higher FRET efficiency was detected in the scattered puncta that might be some vesicle structures, suggesting that the interaction between APP and α-/β-secretase complex in the puncta might be functional. Besides, APP mutants showed different effects on ADAM10/BACE1 BiFC signal, suggesting that the mutations might influence the binding of APP to the protease complex. Moreover, we observed that APP exerted an effect on α-/β-/γ-secretase FRET, indicating that APP might bind to the ternary complex formed by α-/β-/γ-secretase as well, further supporting that the secretases complex might be functional. Nevertheless, the binding of APP to the triple secretase complex needs to be explored further.

## Author Contributions

GP and XW conceptualized and designed the work. XW performed the experiments and analyzed the data. XW and GP wrote the manuscript.

## Conflict of Interest Statement

The authors declare that the research was conducted in the absence of any commercial or financial relationships that could be construed as a potential conflict of interest.
