# Supplementary material for: Visualization of Alzheimer’s Disease Related α-/β-/γ-Secretase Ternary Complex by Bimolecular Fluorescence Complementation Based Fluorescence Resonance Energy Transfer
Source: Front Mol Neurosci. 2018 Nov 27;11:431. doi: 10.3389/fnmol.2018.00431 (PMC6277482; doi:10.3389/fnmol.2018.00431)
Supplement: Supplementary file 1 [file Data_Sheet_1.docx]

Supplementary Material

**Visualization of Alzheimer’s disease related α-/β-/γ-secretase ternary complex by bimolecular fluorescence complementation based fluorescence resonance energy transfer**

**Xin Wang, Gang Pei^*^**

*** Correspondence:** Gang Pei: [gpei@sibs.ac.cn](mailto:gpei@sibs.ac.cn)

##
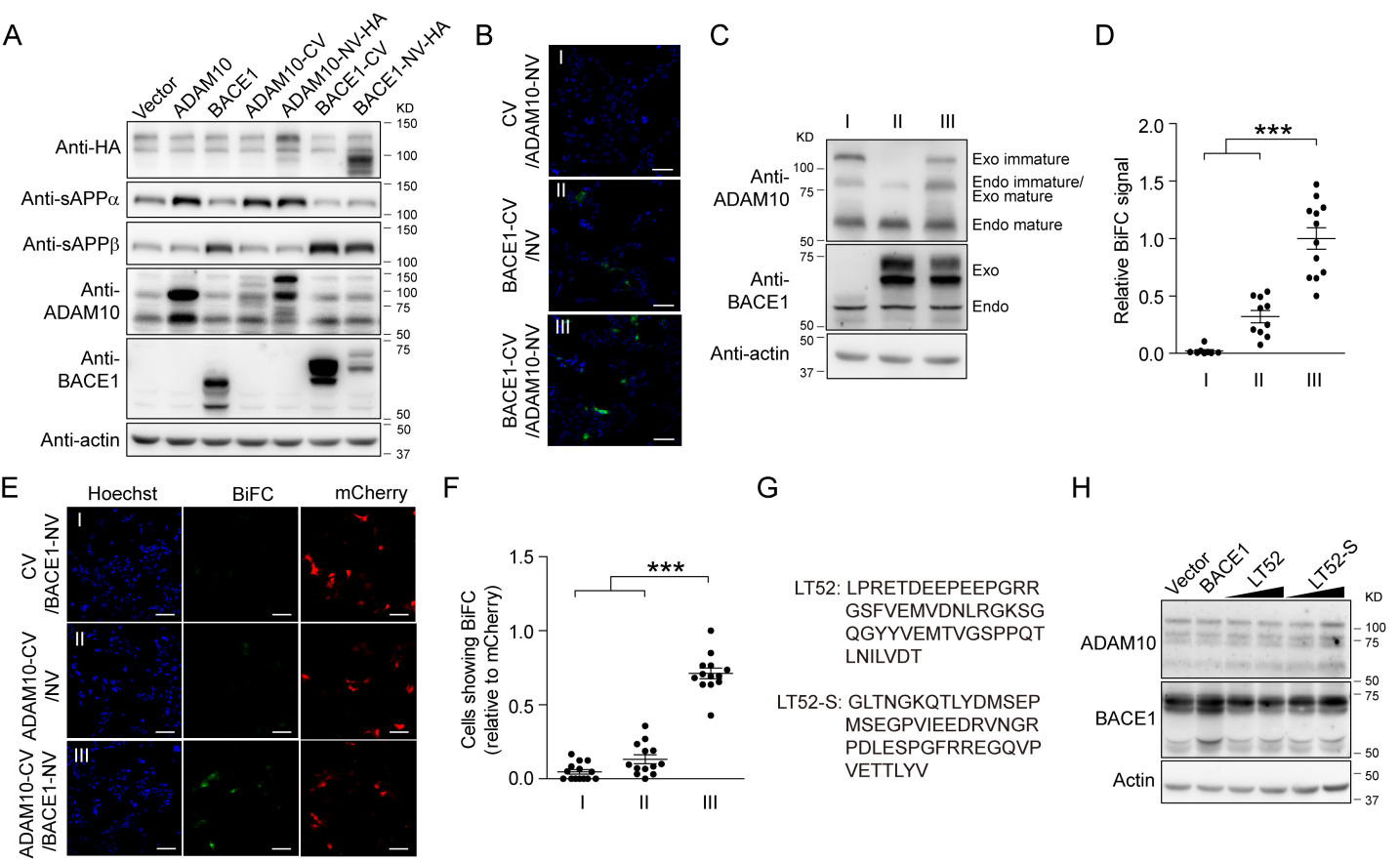
Supplementary Figures

**Supplementary Figure 1 BiFC signal of α-/β-secretase complex.** (A) ADAM10 and BACE1 BiFC probes possess secretase activities in HEK 293 cells stably expressing APPswe-HA. Expression of ADAM10-CV or ADAM10-NV increased the production of sAPPα, while expression of BACE1-CV or BACE1-NV enhanced the generation of sAPPβ. (B) BiFC signal between BACE1-CV and ADAM10-NV. HEK 293 cells were transfected with designated plasmids and fixed 16 hours later. The signals of bimolecular fluorescent Venus were examined by confocal imaging under 20X objective. Scale bar: 100 μm. (C) Western blots show the expression of BACE1-CV and ADAM10-NV in (B). (D) Quantification analysis of fluorescence intensity of BiFC signals in (B). Images under 20X objective were evaluated, N = 8-12 per condition. A one-way ANOVA with Bonferroni’s multiple comparisons was used. *** *p* < 0.001. (E) BiFC signal between BACE1-CV and ADAM10-NV with mCherry expression control. The signals of bimolecular fluorescent Venus and mCherry were examined by confocal imaging under 20X objective. Scale bar: 100 μm. (F) Quantification analysis of BiFC efficiency in (E). Images under 20X objective were evaluated, N = 13-14 per condition. A one-way ANOVA with Bonferroni’s multiple comparisons was used. *** *p* < 0.001. (G) The amino acid sequences of LT52 and LT52-S. (H) Western blots show the expression of BACE1-CV and **
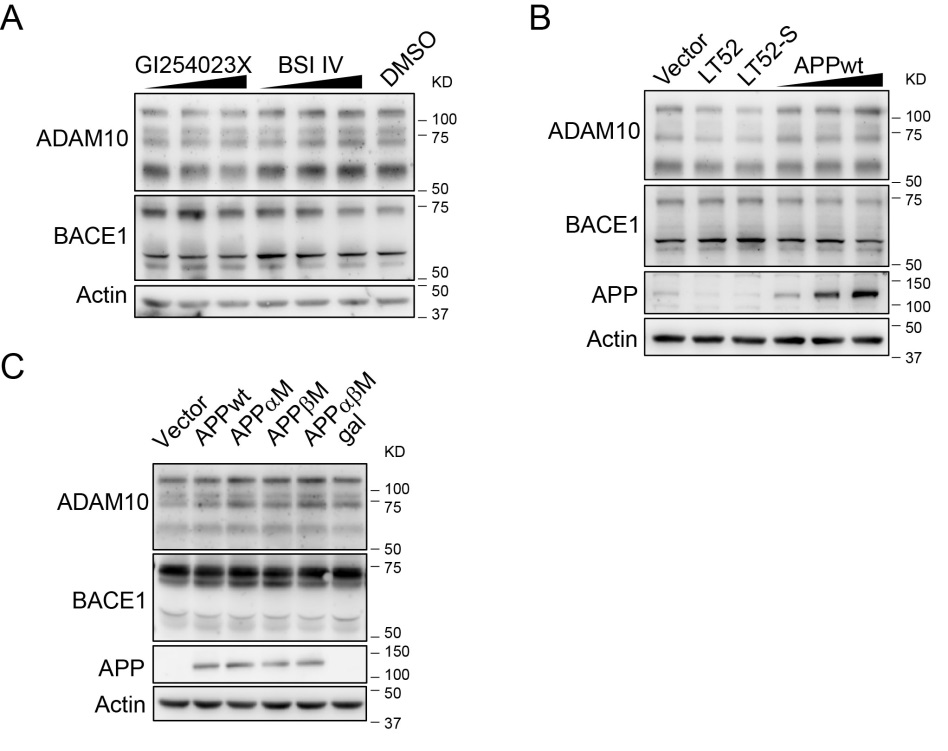
**ADAM10-NV in Figure 1E.

**Supplementary Figure 2 Western blots show the expression of ADAM10 and BACE1 BiFC probes.** (A) Western blots show the expression of ADAM10-CV and BACE1-NV when treated with GI254023X or BSI-IV in Figure 2A. (B) Western blots show the expression of ADAM10-CV, BACE1-NV and APPwt in Figure 2C. (C) Western blots show the expression of ADAM10-CV, BACE1-NV and APP in Figure 2E.


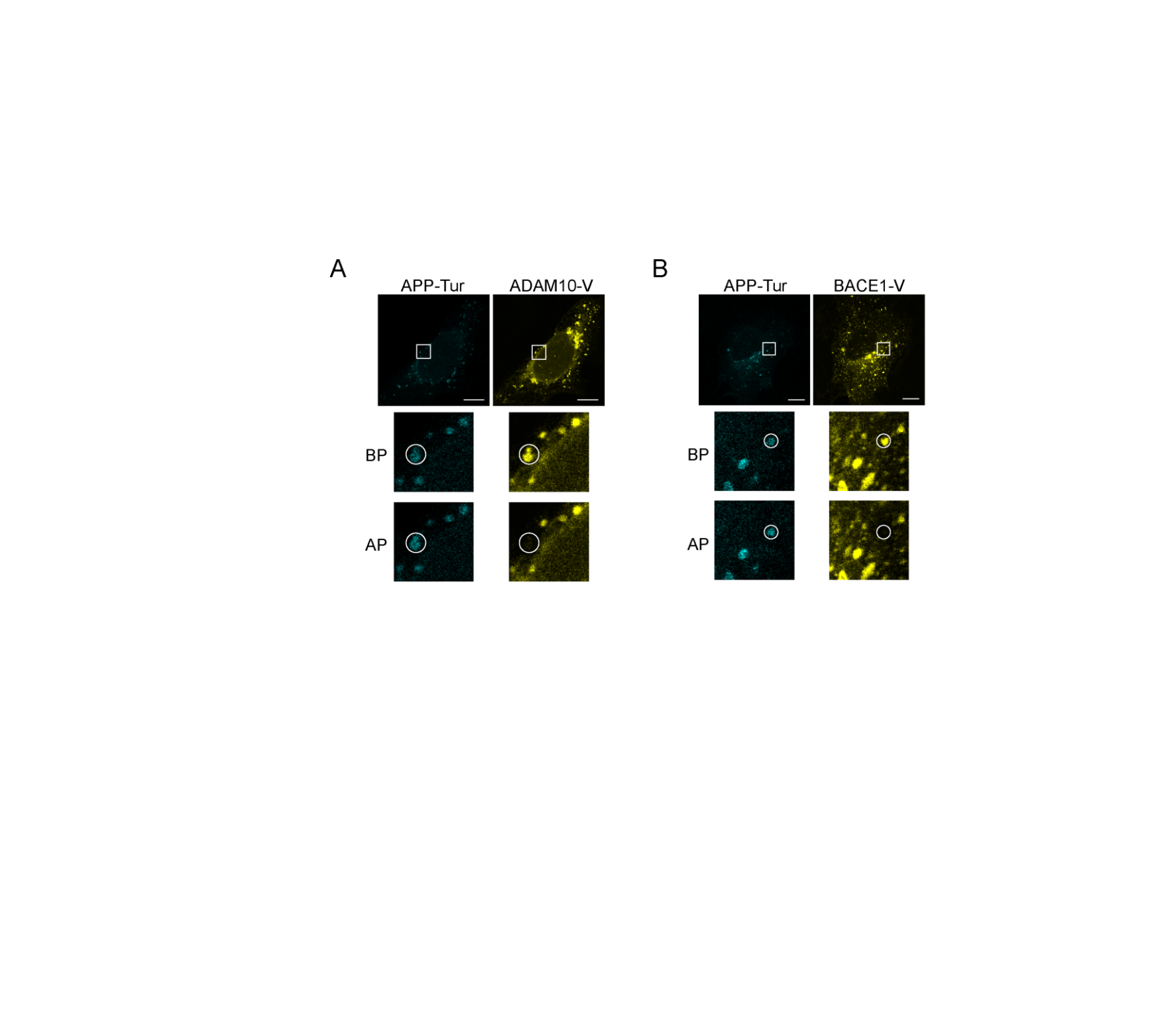


**Supplementary Figure 3 Representative images of FRET analysis between APP and ADAM10 (A), and between APP and BACE1 (B) in Figure 3D.**
